# Exercise Attenuates Intermittent Hypoxia-Induced Cardiac Fibrosis Associated with Sodium-Hydrogen Exchanger-1 in Rats

**DOI:** 10.3389/fphys.2016.00462

**Published:** 2016-10-14

**Authors:** Tsung-I Chen, Wei-Chia Tu

**Affiliations:** ^1^Center of Physical Education, Office of General and Basic Education, Tzu Chi UniversityHualien, Taiwan; ^2^Master program in Physiological and Anatomical Medicine, School of Medicine, Tzu Chi UniversityHualien, Taiwan

**Keywords:** sodium hydrogen exchanger, intermittent hypoxia, fibrosis, oxidative stress, antioxidant capacity, anti-fibrosis, obstructive sleep apnea, heart

## Abstract

**Purpose:** To investigate the role of sodium–hydrogen exchanger-1 (NHE-1) and exercise training on intermittent hypoxia-induced cardiac fibrosis in obstructive sleep apnea (OSA), using an animal model mimicking the intermittent hypoxia of OSA.

**Methods:** Eight-week-old male Sprague–Dawley rats were randomly assigned to control (CON), intermittent hypoxia (IH), exercise (EXE), or IH combined with exercise (IHEXE) groups. These groups were randomly assigned to subgroups receiving either a vehicle or the NHE-1 inhibitor cariporide. The EXE and IHEXE rats underwent exercise training on an animal treadmill for 10 weeks (5 days/week, 60 min/day, 24–30 m/min, 2–10% grade). The IH and IHEXE rats were exposed to 14 days of IH (30 s of hypoxia—nadir of 2–6% O_2_—followed by 45 s of normoxia) for 8 h/day. At the end of 10 weeks, rats were sacrificed and then hearts were removed to determine the myocardial levels of fibrosis index, oxidative stress, antioxidant capacity, and NHE-1 activation.

**Results:** Compared to the CON rats, IH induced higher cardiac fibrosis, lower myocardial catalase, and superoxidative dismutase activities, higher myocardial lipid and protein peroxidation and higher NHE-1 activation (*p* < 0.05 for each), which were all abolished by cariporide. Compared to the IH rats, lower cardiac fibrosis, higher myocardial antioxidant capacity, lower myocardial lipid, and protein peroxidation and lower NHE-1 activation were found in the IHEXE rats (*p* < 0.05 for each).

**Conclusion:** IH-induced cardiac fibrosis was associated with NHE-1 hyperactivity. However, exercise training and cariporide exerted an inhibitory effect to prevent myocardial NHE-1 hyperactivity, which contributed to reduced IH-induced cardiac fibrosis. Therefore, NHE-1 plays a critical role in the effect of exercise on IH-induced increased cardiac fibrosis.

## Introduction

Intermittent hypoxia (IH) is a significant symptom in patients with obstructive sleep apnea (OSA) and is caused by recurrent upper airway collapse during sleep (Filgueiras-Rama et al., 2013). IH in OSA is a major factor in left ventricle (LV) remodeling, cardiac hypertrophy, fibrosis and ventricular dysfunction, leading to impaired cardiac contractility, and heart failure (Baguet et al., 2012; Yin et al., 2012). Evidence is now emerging that sodium-hydrogen exchanger-1 (NHE-1) contributes to chronic maladaptive myocardial responses to injury, such as post-infarction myocardial remodeling and likely contributes to the development of heart failure (Karmazyn et al., 2008). However, whether or not IH-induced heart disease is associated with NHE-1 remains unknown.

Sodium-hydrogen exchange is the primary process by which the cardiac cell extrudes protons, particularly under conditions of intracellular acidosis (Karmazyn et al., 2008; Xue et al., 2010; Feger and Starnes, 2013a). Pharmacological evidence in support of the beneficial effects of NHE-1 inhibition in ischemia/reperfusion injury has been confirmed in mouse hearts with genetic ablation of NHE-1 with enhanced resistance to ischemic and reperfusion injury (Cingolani and Ennis, 2007). During ischemia/reperfusion, dramatically increased levels of reactive oxygen species (ROS) damage the NHE-1 regulatory capacity; this impairs cardiomyocyte acidification with failure to preserve intracellular pH (Li et al., 2007).

In addition, NHE-1 phosphorylation plays a role in the process by which lipopolysaccharide or oxygen and glucose deprivation and reoxygenation treatments trigger microglial proinflammatory responses (Liu et al., 2010). Even low levels of ROS can enhance NHE1 activity in rat hearts (Feger and Starnes, 2013b). However, NHE-1 hyperactivity causes a high concentration of intracellular Na^+^ that accelerates sodium-calcium exchange and results in intracellular Ca^2+^ overload, leading to ventricular dysfunction, cardiac hypertrophy, apoptosis, and heart failure (Besse et al., 2004; Cingolani and Ennis, 2007). Although it is known that IH-induced increased levels of ROS can increases NHE-1 expression (Chen et al., 2015), whether or not NHE-1 hyperactivity plays a role in IH-induced cardiac fibrosis remains unclear.

On the other hand, strenuous endurance exercise can enhance the NHE-1 regulatory capacity (Feger and Starnes, 2013b) to maintain intracellular pH and NHE-1 expression under normal physiological conditions and to attenuate H_2_O_2_-induced NHE-1 hyperactivity in isolated cardiomyocytes (Feger and Starnes, 2013a). Although exercise attenuates NHE-1 protein content and provides beneficial effects against IH-induced LV impairment (Chen et al., 2015), whether or not these effects play a role in alleviating IH-induced cardiac fibrosis remains unknown. Therefore, the purpose of this study was to investigate the role of NHE-1 in exercise-induced attenuation of cardiac fibrosis in patients with OSA using an animal model mimicking the IH of OSA.

## Methods

### Animal preparation

Six-week-old, male, Sprague–Dawley rats were purchased from BioLASCO Taiwan Co. Ltd. The rats were maintained on an artificial 12-h light–dark cycle in the Laboratory Animal Center, Tzu Chi University. Water and food were available *ad libitum*. All surgical and experimental procedures were conducted using recommended procedures approved by the Institutional Animal Care and Use Committee of Tzu Chi University.

After 2 weeks of feeding, the rats were randomly assigned to a control (CON) group or an exercise (EXE) group from weeks 1–10. At weeks 9–10, the CON rats were randomly divided into CON exposed to normal air (CON) or intermittent hypoxia (IH) groups, and the EXE rats were randomly divided into EXE exposed to normal air (EXE) or IH (IHEXE) groups. The CON, IH, EXE, and IHEXE groups were randomly assigned to subgroups receiving either N-Aminoiminomethyl-4-1-Methylethyl-3-Methylsulfonylbenzamide (cariporide, Sigma, MO, USA), which is an NHE-1 blocker, or 0.9% NaCl (vehicle) for 14 consecutive days. In this study, rats received either 1 mg/kg of cariporide dissolved in 0.9% NaCl or an equal amount of vehicle. Cariporide is a highly selective NHE-1 inhibitor with no apparent effects on the Na^+^/Ca^2+^ exchanger or fast Na^+^ currents at levels not exceeding 10 μmol/l (Kolarova et al., 2005). Several NHE-1 inhibitors have been developed with the goal of cardioprotection after exchanger activation by the prevention of [Na^+^]_i_ and [Ca^2+^]_i_ accumulation. Amiloride, a K^+^-sparing diuretic, was the first compound that was proposed to inhibit the exchanger. NHE-1 and NHE-2 are the isoforms that are most sensitive to this drug, which also inhibits Na^+^ channels and NCX (Cingolani and Ennis, 2007). Therefore, cariporide was used in this study because it specifically inhibits the NHE-1 exchanger.

### Intermittent hypoxia

**Figure 1:**
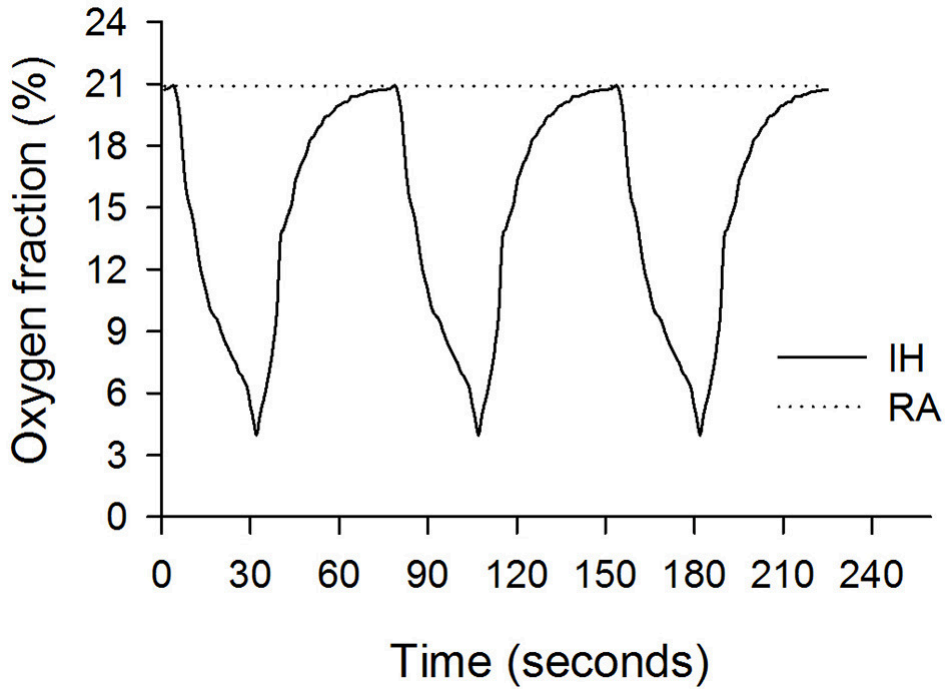
**Oxygen fractions during intermittent hypoxia (IH, solid line) and room air (RA, dashed line) recorded in the Plexiglas chambers**.

The IH process has been described in our previous studies (Chen et al., 2011a,b, 2015). In brief, the rats were housed in Plexiglas cylindrical chambers with snug-fitting lids. Using a timed solenoid valve, pure nitrogen was distributed to the chambers to reduce the ambient inspired O_2_ fraction gradually to 2%–6% at the nadir, and it remained at a low nadir for 2–5 s once per 30 s. This was followed by infusion of compressed air (for ~45 s), allowing the gradual return of ambient air to enable an inspired O_2_ fraction of 20.9%. The duration of each repetitive hypoxia–reoxygenation cycle was 75 s and 48 cycles per hour (Figure 1). The rats were exposed to IH for 8 h per day during the daytime for 14 consecutive days.

### Exercise protocol

The exercise protocol was modified from previous studies (Siu et al., 2004; Thorp et al., 2007). In brief, animals assigned to the exercise groups were habituated to treadmill exercise at a speed of 10 m/min for 5 consecutive days, involving a gradual increase in running speed, beginning with 12 m/min and ending with 24 m/min. After a 2-day rest, the animals performed 10 weeks of treadmill exercise. In the first week, the animals ran on the treadmill at a speed of 24 m/min on a 2% grade, involving a gradual increase in running time, beginning with 20 min per day and ending with 60 min per day. At weeks 2–10, the animals ran for 60 min per day on a 2–10% grade. At weeks 1–5, the speed of the treadmill was gradually increased by 2 m/min per week up to 30 m/min. At weeks 6–10, the grade of the treadmill was gradually increased by 2% per week up to 10% at a speed of 30 m/min (Table 1). The exercise-training rats warmed up and cooled down on the treadmill at a speed of 15 m/min on a 0% grade for 5 min in each bout of exercise.

**Table 1:** **Exercise training protocol of the rats**.

	**Weeks**									
	**0**	**1**	**2**	**3**	**4**	**5**	**6**	**7**	**8**	**9–10**
Duration (min)	10	20–60	60	60	60	60	60	60	60	60
Speed (m/min)	12–24	24	24	26	28	30	30	30	30	30
Incline (%)	2	2	2	2	2	2	4	6	8	10

### Myocardial tissue preparation

All rats were sacrificed at the same time point, i.e., 24 h after the last bout of IH exposure, with two intraperitoneal injections of 750 mg/kg urethane (Sigma, St. Louis, MO, USA) in a 10-min interval. The following procedures have been described in our previous studies (Chen et al., 2011a,b, 2015). In brief, following a midline skin incision, the chest plate was reflected in order to expose the heart and lungs. The heart was lifted slightly and removed by cutting the aortic arch beyond the left subclavian artery (1.0–1.5 cm from the heart). The heart was immediately arrested and immersed in ice-cold phosphate-buffered saline (PBS) solution. After the heart stopped beating, it was removed from the PBS, and the aorta was immediately affixed to a cannula attached to a syringe. The hearts were subjected to retrograde perfusion (pressure: 110 mmH_2_O) with PBS for 1 min to wash out blood. The LV myocardium was quickly removed and divided into six sections. The third section counted from the apex was soaked in Bouin's solution (Sigma, MO, USA). The other sections were snap frozen in liquid nitrogen and stored at −80°C until use. Care was taken to harvest the same region of the LV myocardium from all animals consistently.

### Cardiac fibrosis index

Hearts were fixed in Bouin's solution overnight, embedded in paraffin and serially sectioned into 5-μm slices. Samples were stained with haematoxylin-eosin (HE) to evaluate gross morphology or Masson's trichrome to assess fibrosis. The cardiac fibrosis index was quantified using Sirius red stain (Liu et al., 2012).

### Real-time quantitative polymerase chain reaction (Q-PCR)

This protocol was adapted and modified from a previous study (Guo et al., 2011). In brief, total RNA was isolated using the RNeasy Mini Kit and RNase-Free DNase Set (Qiagen) and reverse transcribed using the Omniscript RT Kit (Qiagen), as described previously. The following primers were used:

Collagen-1: forward primer, 5′-G GTGCTACATCTCTGTGAAG 3′ and reverse primer, 5′- AAAGGAACATTTGCTGGTCTA 3′;
Fibronectin: forward primer, 5′-CT GCCGAATGTAGATGAGGA-3′ and reverse primer, 5′- ATGAGGATAGAGGTGGTAGTCT-3′;
NHE-1: forward primer, 5′-A CATTCAACAGTGGAGTGACT-3′ and reverse primer, 5′- TGGCAGGGAAGATTCAAAGG-3′; and
GAPDH: forward primer, 5′-TG CACCACCAACTGCTTAGC-3′ and reverse primer, 5′- GCCCCACGGCCA TCA-3′.

PCR was performed using the SYBR Green method with TaqDNA polymerase (Invitrogen). Fluorescence data were acquired at the end of extension. The cycle threshold value was measured and calculated using computer software (LightCycler 480 II, Roche, USA). We used 3 μg of total RNA to perform each reverse transcription. A 1:10 dilution of cDNA obtained in the RT reaction (25 μL total volume) was used in each qPCR. The comparative C_t_ method (2^−ΔΔCt^) was used to quantify gene expression, where ΔΔC_t_ = ΔC_t_ (sample) −ΔC_t_ (reference).

### Western blot

This protocol was adapted and modified from our previous studies (Chen et al., 2011a,b). Total cytosolic fraction extracts were obtained from the LV myocardium using a commercially available isolation kit (Pierce, Rockford, IL, USA), according to the manufacturer's instructions. Total protein content was quantified in duplicates using a protein assay kit (Bio-Rad, Hercules, CA, USA) and bovine serum albumin (Sigma) standards. Samples (30 μg) were resolved on 4–16% gradient SDS–PAGE for 2 h at room temperature and then electrophoretically transferred onto polyvinylidine fluoride (PVDF) membranes (Amersham, Piscataway, NJ, USA). The membranes were blocked with 5% non-fat milk in Tris-buffered saline with 0.05% Tween 20 (TBS-T) at room temperature for 1 h and probed with primary antibodies for anti-TIMP-1 (1:200, Santa Cruz, CA, USA), anti-TGF-β (1:1000, Cell Signaling, MA, USA) and anti-MMP-9 (1:1000, Abcam, MA. USA), anti-NHE-1 (1:1000, Chemicon), anti-phospho-(Serine) 14-3-3 binding motif (BM 14-3-3) (1:1000, Cell Signaling, MA, USA) and GAPDH (1:2000, Santa Cruz, CA, USA), diluted in TBS-T with 2% BSA. All primary antibody incubations were conducted at 4°C overnight. The membranes were then incubated with horseradish peroxidase-conjugated secondary antibodies at room temperature for 1 h, and the signals were developed by enhanced chemiluminescence (Amersham, GE Healthcare, Buckinghamshire, UK) The signals were visualized using a UVP BioSpectrum 810 (Analytik Jena AG, Jena, Germany). The resultant bands were captured by a scanner and quantified as arbitrary units (OD × band area) by ImageJ analysis software (National Institutes of Health, Bethesda, MD, USA). Protein expression in the LV myocardium was reported as the ratio of protein to GAPDH.

### Myocardial oxidative stress and antioxidant capacity

Oxidative stress was determined by measuring myocardial levels of lipid and protein peroxidation. Lipid peroxidation was determined by measuring myocardial levels of malondialdehyde (MDA) using a competitive enzyme immunoassay with a plate reader (Thermo Scientific Multiskan Spectrum, Rockford, IL, USA). Measurements were obtained using a commercially available lipid peroxidation colorimetric assay kit (BioVision, Milpitas, CA, USA), according to the manufacturer's instructions. Protein carbonyl content was determined by the reaction between 2,4-dinitrophenylhydrazine (DNPH) and protein carbonyls, forming a Schiff base with a plate reader. Measurements were obtained using a commercially available protein carbonyl colorimetric assay kit (Cayman Chemical, Ann Arbor, MI, USA), according to the manufacturer's instructions. All measurements were performed in duplicate on the same microtiter plate with the same setting. The amount of MDA and protein carbonyl was normalized to the total amount of myocardium in the sample and calculated in nmol/mg protein.

Antioxidant capacity was determined by measuring catalase and superoxide dismutase (SOD) activities. Catalase activity was determined by using a formaldehyde solution as standard. Measurements were obtained using a commercially available catalase activity colorimetric assay kit (BioVision, Milpitas, CA, USA). Total SOD activity assay utilizes a tetrazolium salt for detection of superoxide radicals generated by xanthine oxidase and hypoxanthine using a commercially available SOD assay kit (Cayman Chemical, Ann Arbor, MI, USA). All measurements were performed in duplicate on the same microtiter plate in the same setting, according to the manufacturer's instructions. The unit of catalase and total SOD activities was U/mg protein.

### The citrate synthase activity of soleus

Measurements were obtained using a commercially available citrate synthase activity colorimetric assay kit (BioVision, Milpitas, CA, USA), according to the manufacturer's instructions. The absorbance of standard and samples was read with a plate reader (Thermo Scientific, Rockford, IL, USA). All measurements were performed in duplicate on the same microtiter plate in the same setting. The total citrate acid synthase activity was normalized to the total amount of myocardium in the sample and calculated in U/mg protein.

### Statistical analyses

Statistical analyses were performed using SPSS 13.0 software (SPSS, Inc. Chicago, IL, USA). All values are expressed as means and standard errors of means. Body weight during exercise training from week 0 to 8 was analyzed using two-way analysis of variance (ANOVA) to assess main effects for groups (CON and EXE) and time (week 0–8) as the independent and repeated variables, respectively. Body weight during IH exposure from day 0 to 14 was analyzed using three-way ANOVA to assess main effects for groups (CON, IH, EXE, and IHEXE), treatments (Veh and cariporide) and time (day 0 and 14) as the independent (groups and treatments) and repeated variables (time). Citrate synthase activity of soleus, myocardial levels of MDA, and protein carbonyl, myocardial catalase and total SOD activity and myocardial levels of TGF-β, TIMP-1, MMP-9, NHE-1, BM 14-2-2, and GAPDH at week 10 were analyzed using two-way ANOVA to assess main effects for groups (CON, IH, EXE, and IHEXE) and treatments (Veh and cariporide) as the independent variables. Tukey's honestly significant difference (HSD)-protected least-significant difference test was used to determine differences between the means when comparing more than two groups. The level of significance was set at *p* < 0.05 for all analyses.

## Results

### Body weight

**Figure 2:**
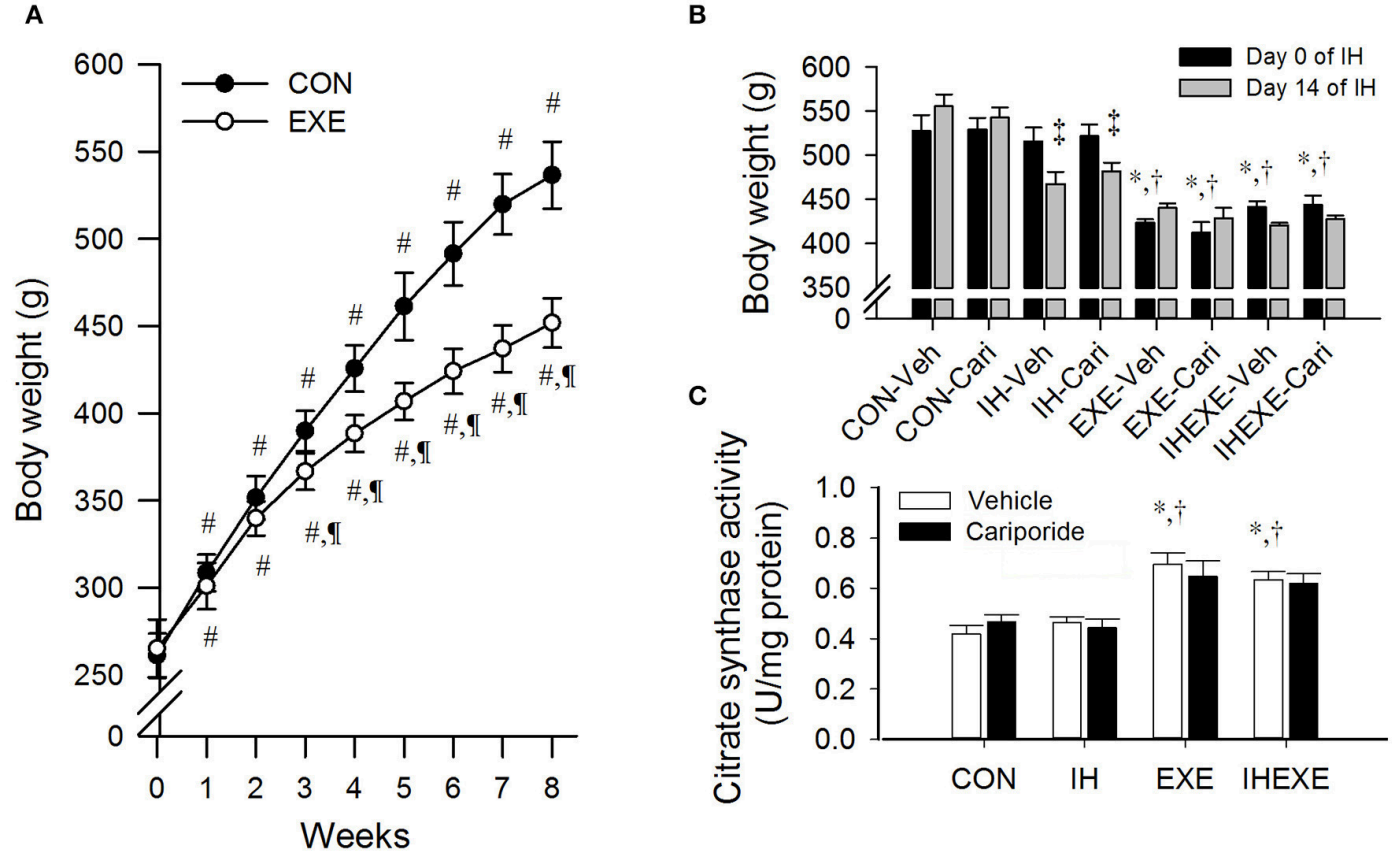
**The effects of exercise on body weight at weeks 1–8 (***n*** = 20 animals per group) (A), intermittent hypoxia on body weight at weeks 9–10 (***n*** = 5 animals per group) (B) and exercise on citrate synthase activity (***n*** = 5 animals per group) (C)**. ^#^*p* < 0.05 compared to week 0; ^¶^*p* < 0.05 compared to controls (CON); ^*^p < 0.05 compared to controls receiving vehicle (CON-Veh); ^†^*p* < 0.05 compared to IH receiving vehicle (IH-Veh); ^‡^*p* < 0.05 compared to day 0 in each group; Veh, vehicle; Cari, cariporide; IH, intermittent hypoxia; IHEXE, intermittent hypoxia combined with exercise.

The body weight significantly increased from 15 to 51% in the controls and from 12 to 41% in the exercise group from week 1 to 8, respectively, compared to week 0 (all *p* < 0.05; Figure 2A). Compared to the control group, body weight was significantly lowered from 6 to 19% in the exercise group from week 3 to 8 (all *p* < 0.05; Figure 2A). During the 14 days of IH exposure, the body weight of the EXE-Veh and IHEXE-Veh groups were significantly lowered by 25 and 20%, respectively, compared to the CON-Veh group at day 0 (all *p* < 0.05; Figure 2B). The body weight of the EXE-Veh and IHEXE-Veh groups were significantly lowered by 22 and 17%, respectively, compared to the IH-Veh group at day 0 (all *p* < 0.05; Figure 2B). At day 14, the body weight of the IH-Veh and IH-cari groups were significantly decreased by 10 and 8%, respectively, compared to day 0 (all *p* < 0.05; Figure 2B). There were no significant differences in body weight between the IH-Veh group and the IH-cari group or between the IHEXE-Veh group and the IHEXE-cari group after 14 days of IH (all *p* > 0.05; Figure 2B).

### The citrate synthase activity of soleus

The citrate synthase activity of soleus in the EXE-Veh and IHEXE-Veh groups were significantly higher by 40 and 34%, respectively, compared to the CON-Veh group, and 33 and 27%, respectively, compared to the IH-Veh group (all *p* < 0.05; Figure 2C). However, the citrate synthase activity in the IH-Veh group did not differ significantly from that in the CON-Veh group (*p* > 0.05; Figure 2C). Furthermore, there were no significant differences in citrate synthase activity between the CON-Veh and CON-Cari groups, the IH-Veh and IH-Cari groups, the EXE-Veh and EXE-Cari groups and the IHEXE-Veh and IHEXE-Cari groups (all *p* > 0.05; Figure 2C).

### Cardiac fibrosis index

**Figure 3:**
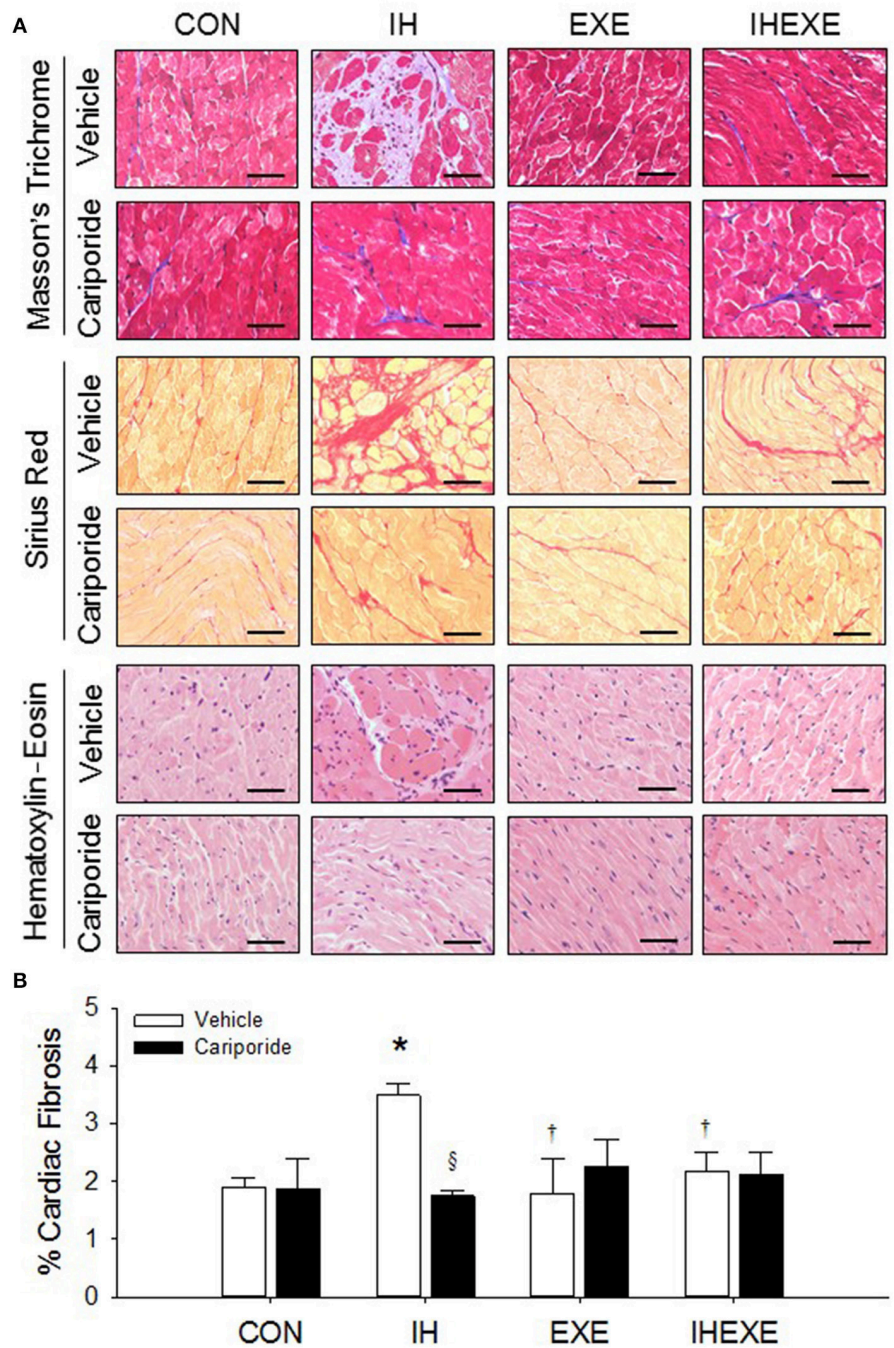
**The effect of exercise and cariporide on IH-induced cardiac fibrosis evaluated by Masson's trichrome, Sirius red and Haematoxylin–Eosin stains (***n*** = 5 animals per group) (A)**. The cardiac fibrosis index was quantified by using Sirius red stain (*n* = 5 animals per group) **(B)**. ^*^p < 0.05 compared to controls receiving vehicle (CON-Veh); ^†^*p* < 0.05 compared to IH receiving vehicle (IH-Veh); ^§^*p* < 0.05 compared to vehicle in each group; EXE, exercise; IHEXE, IH combined with EXE. Scale bar = 50 μm.

**Figure 4:**
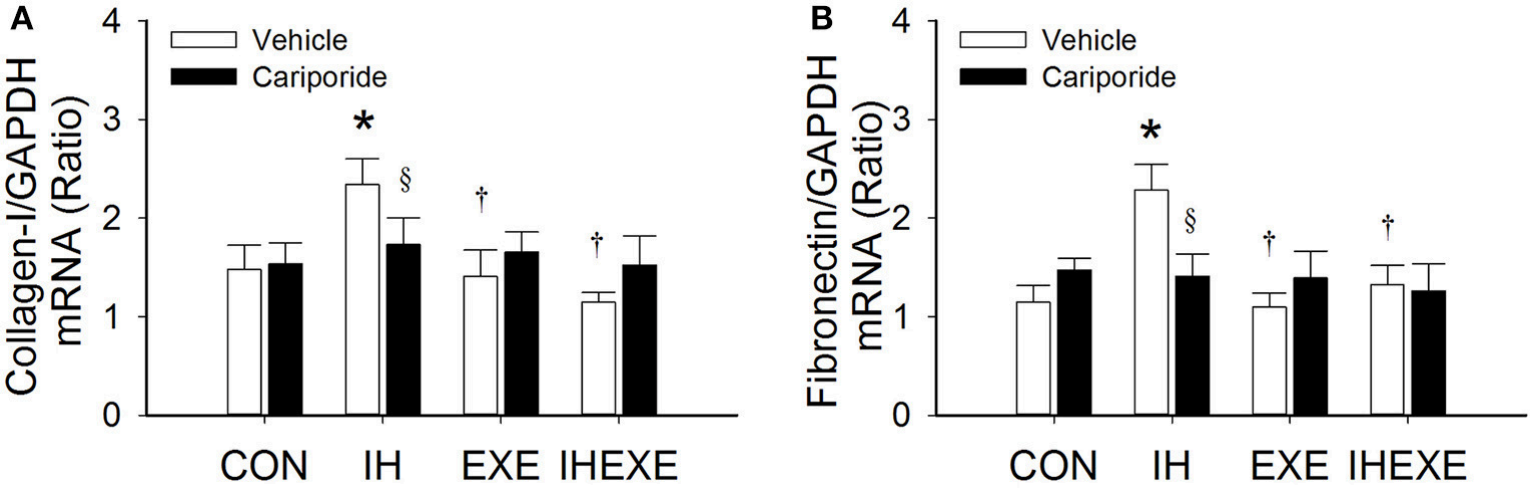
**The effect of exercise and cariporide on IH-induced myocardial expression of collagen-1 (A) and fibronectin (B) mRNA (***n*** = 5 animals per group)**. ^*^p < 0.05 compared to controls receiving vehicle (CON-Veh); ^†^*p* < 0.05 compared to IH receiving vehicle (IH-Veh); ^§^*p* < 0.005 compared to vehicle in each group; EXE, exercise; IHEXE, IH combined with EXE.

IH increased cardiac fibrosis as determined by Masson's trichrome and haematoxylin-eosin stains (Figure 3A). The cardiac fibrosis index was calculated using Sirius red stain. The results showed that the cardiac fibrosis index was significantly higher by 45% in the IH-Veh group compared to the CON-Veh group (*p* < 0.05; Figure 3B). Compared to the CON-Veh group, there were no significant differences in the cardiac fibrosis index in the EXE-Veh group and the IHEXE-Veh group (both *p* > 0.05; Figure 3B). However, the cardiac fibrosis index was significantly lower by 94 and 61% in the EXE-Veh and IHEXE-Veh groups, respectively, compared to the IH-Veh group (both *p* < 0.05; Figure 3B). Compared to the subgroups that received a vehicle, cariporide attenuated the cardiac fibrosis in the IH group (all *p* < 0.05; Figure 3B). Moreover, there were no significant differences in the levels of the cardiac fibrosis index between the CON-Cari and the CON-Veh groups, the EXE-Cari and the EXE-Veh groups and the IHEXE-Cari and the IHEXE-Veh groups (*p* > 0.05; Figures 3A,B). In addition, the myocardial expression of collagen-1 and fibronectin mRNA was significantly higher by 37 and 50%, respectively, in the IH-Veh group compared to the CON-Veh group (both *p* < 0.05; Figures 4A,B). There were no significant differences in collagen-1 and fibronectin mRNA in the EXE-Veh and IHEXE-Veh groups compared to the CON-Veh group (both *p* > 0.05; Figures 4A,B). Compared to the IH-Veh group, collagen-1 and fibronectin mRNA were significantly lower by 66 and 104%, respectively, in the EXE-Veh group, and by 107 and 72%, respectively, in the IHEXE-Veh group (all *p* < 0.05; Figures 4A,B). Compared to the subgroups that received a vehicle, cariporide attenuated the myocardial expression of collagen-1 mRNA by 35% and fibronectin mRNA by 62% in the IH group (all *p* < 0.05; Figures 4A,B). However, there were no significant differences between the CON-Cari and CON-Veh groups, the EXE-Cari and the EXE-Veh groups and the IHEXE-Cari and IHEXE-Veh groups (all *p* > 0.05; Figures 4A,B).

### Myocardial expression of TGF-β, TIMP-1 and MMP-9

**Figure 5:**
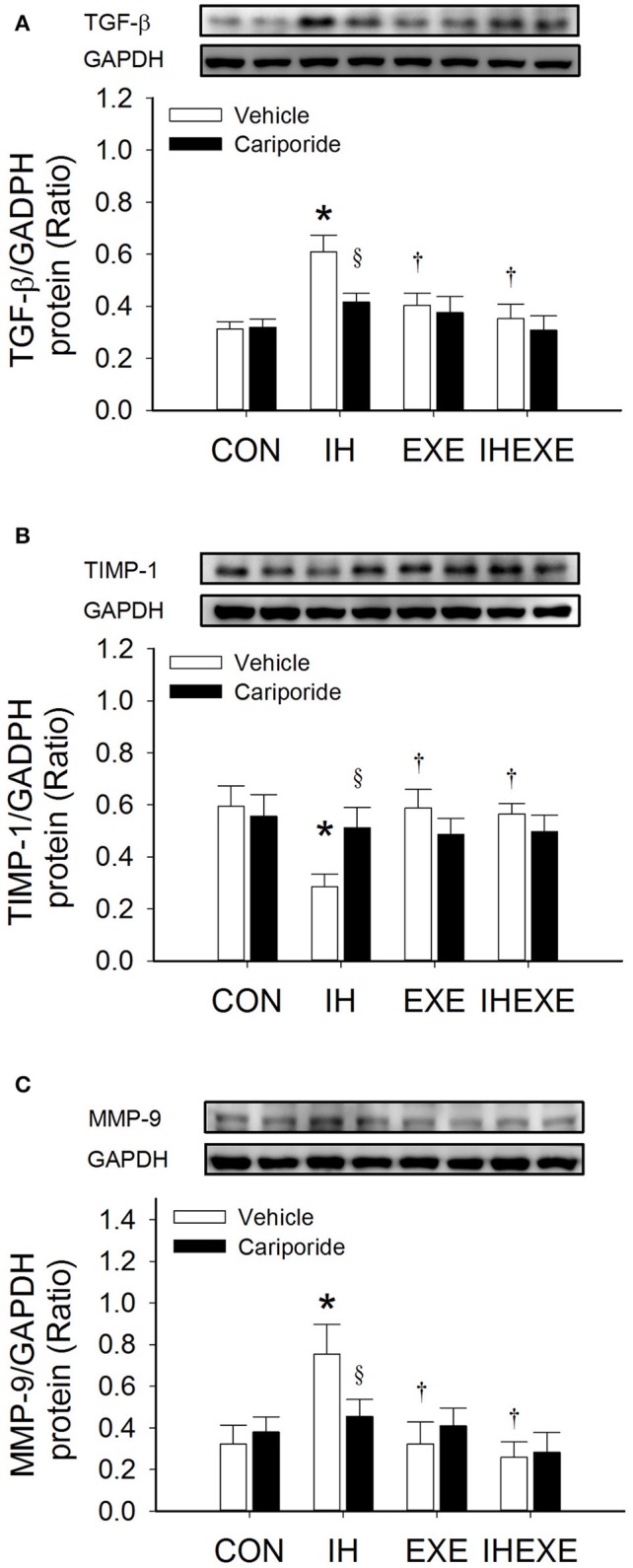
**The effect of exercise and cariporide on IH-induced myocardial expression of TGF-β (A), TIMP-1 (B) and MMP-9 protein (C) (***n*** = 5 animals per group)**. ^*^p < 0.05 compared to controls receiving vehicle (CON-Veh); ^†^*p* < 0.05 compared to IH receiving vehicle (IH-Veh); ^§^*p* < 0.05 compared to vehicle in each group; EXE, exercise; IHEXE, IH combined with EXE.

Compared to the CON-Veh group, the myocardial expression of TGF-β and MMP-9 proteins was significantly increased by 49 and 57%, respectively, and the myocardial expression of TIMP-1 protein was significantly decreased by 109% in the IH-Veh group (all *p* < 0.05; Figures 5A–C). However, there were no significant differences in TGF-β, MMP-9 and TIMP-1 expressions in the EXE-Veh and the IHEXE-Veh groups (both *p* > 0.05; Figures 5A–C) compared to the CON-Veh group. Compared to IH-Veh group, the myocardial expression of TGF-β and MMP-9 proteins were significantly lower by 50 and 134%, respectively, and TIMP-1 protein was significantly higher by 52% in the EXE-Veh group (all *p* < 0.05; Figures 5A–C). The myocardial expression of TGF-β and MMP-9 proteins were significantly lower by 72 and 189%, respectively, and TIMP-1 protein was significantly higher by 50% in the IHEXE-Veh group compared to the IH-Veh group (all *p* < 0.05; Figures 5A–C). Compared to IH-Veh, cariporide decreased the myocardial expression of TGF-β protein by 46% and MMP-9 protein by 65% and increased the TIMP-1 protein by 44% in the IH group (all *p* < 0.05; Figures 5A–C). However, there were no significant differences between the CON-Cari and CON-Veh groups, the EXE-Cari and EXE-Veh groups and the IHEXE-Cari and IHEXE-Veh groups (all *p* > 0.05; Figures 5A–C).

### Myocardial oxidative stress

**Figure 6:**
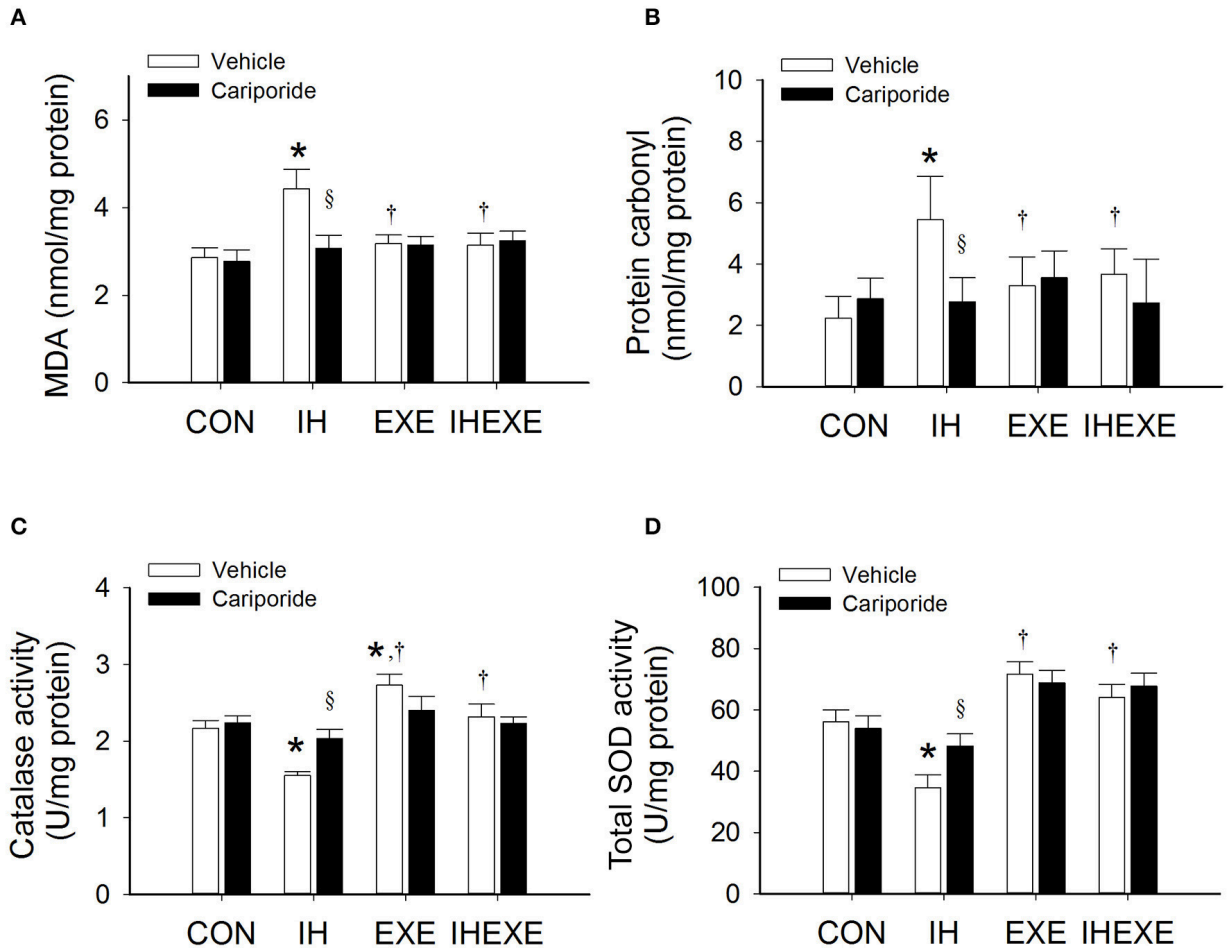
**The effect of exercise and cariporide on IH-induced myocardial MDA (A), protein carbonyl (B), catalase activity (C) and total SOD activity (D) (***n*** = 5 animals per group)**. ^*^p < 0.05 comared to controls receiving vehicle (CON-Veh); ^†^*p* < 0.05 compared to IH receiving vehicle (IH-Veh); ^§^*p* < 0.05 compared to vehicle in each group; EXE, exercise; IHEXE, IH combined with EXE.

In the IH-Veh group, the lipid peroxidation (MDA) and protein carbonyl was significantly higher by 36 and 59% (*p* < 0.05; Figures 6A,B), respectively, when compared to the CON-Veh group. However, there was no significant difference in the level of lipid and protein peroxidation between the IHEXE-Veh and the CON-Veh groups (both *p* > 0.05; Figures 6A,B). In the EXE-Veh and IHEXE-Veh groups, there was significantly lower lipid peroxidation by 39 and 41%, respectively, and significantly lower protein peroxidation by 66 and 49%, respectively, in the LV compared to the IH-Veh group (all *p* < 0.05; Figures 6A,B). Compared to the subgroups that received a vehicle, cariporide decreased lipid and protein peroxidation in the LV in the IH group (all *p* < 0.05; Figures 6A,B); however, there were no significant differences between the CON-Cari and CON-Veh groups, the EXE-Cari and EXE-Veh groups, and the IHEXE-Cari and IHEXE-Veh groups (all *p* > 0.05; Figures 6A,B).

### Myocardial antioxidative capacities

In the IH-Veh group, the myocardial catalase and total SOD activity was significantly lower by 40 and 62% (*p* < 0.05; Figures 6C,D), respectively, when compared to the CON-Veh group. In the EXE-Veh group, the catalase activity was significantly higher by 21% compared to the CON-Veh group (*p* < 0.05; Figure 6C). However, there was no significant difference in the total SOD activity between the EXE-Veh and the CON-Veh groups (both *p* > 0.05; Figure 6D). In the EXE-Veh and IHEXE-Veh groups, there was significantly higher catalase activity by 43 and 33%, respectively, and significantly higher total SOD by 52 and 46%, respectively, in the LV compared to the IH-Veh group (all *p* < 0.05; Figures 6C,D). Compared to the subgroups that received a vehicle, cariporide increased catalase and total SOD activity in the LV in the IH group (all *p* < 0.05; Figures 6C,D); however, there were no significant differences between the CON-Cari and CON-Veh groups, the EXE-Cari and EXE-Veh groups, and the IHEXE-Cari and IHEXE-Veh groups (all *p* > 0.05; Figures 6C,D).

### Myocardial expression of NHE-1 and BM 14-3-3

**Figure 7:**
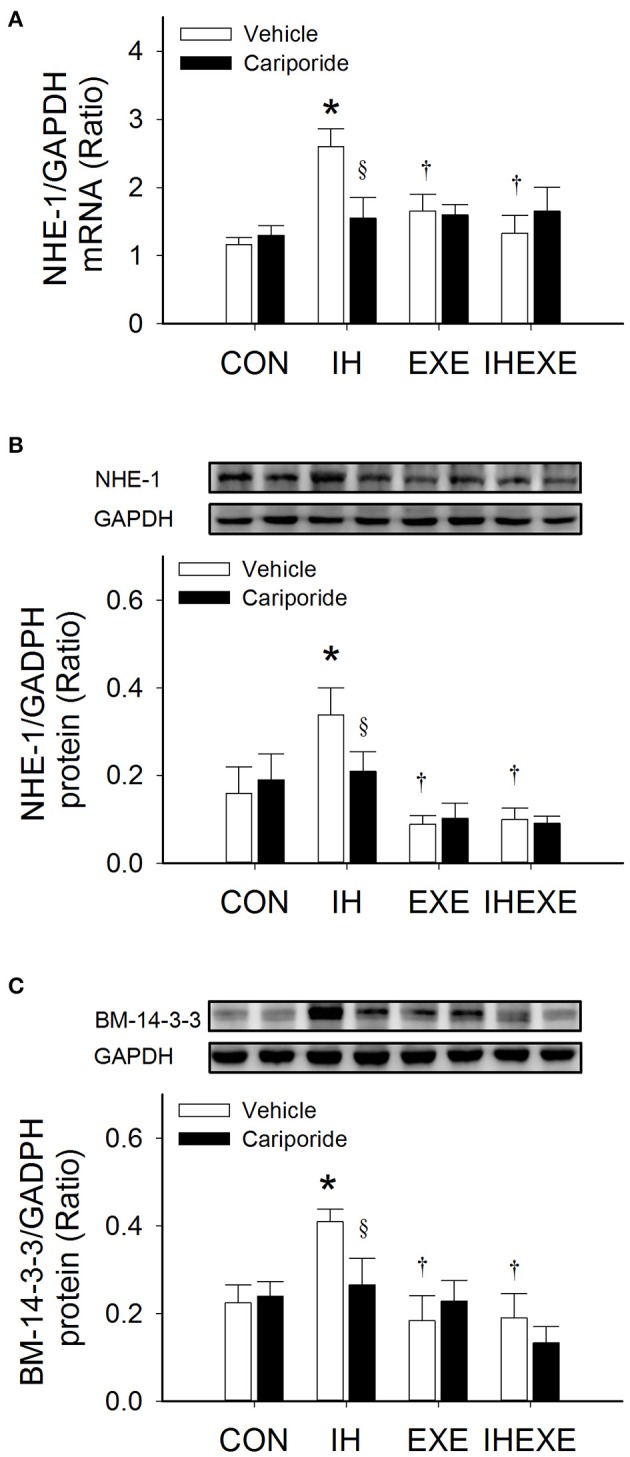
**The effect of exercise and cariporide on IH-induced myocardial expression of NHE-1 mRNA (A), NHE-1 protein (B) and BM 14-3-3 protein (C) (***n*** = 5 animals per group)**. ^*^p < 0.05 compared to controls receiving vehicle (CON-Veh); ^†^*p* < 0.05 compared to IH receiving vehicle (IH-Veh); ^§^*p* < 0.05 compared to vehicle in each group; EXE, exercise; IHEXE, IH combined with EXE.

Compared to the CON-Veh group, the myocardial expression of NHE-1 and BM 14-3-3 proteins and NHE-1 mRNA was significantly increased by 53, 45, and 55%, respectively, in the IH-Veh group (all *p* < 0.05; Figures 7A–C). However, there were no significant differences in NHE-1 and BM 14-3-3 proteins and NHE-1 mRNA expressions in the EXE-Veh and the IHEXE-Veh groups (both *p* > 0.05; Figures 7A–C) compared to the CON-Veh group. Compared to IH-Veh group, the myocardial expression of NHE-1 and BM 14-3-3 proteins and NHE-1 mRNA was significantly lower by 281, 123, and 57%, respectively, in the EXE-Veh group (all *p* < 0.05; Figures 7A–C). The myocardial expression of NHE-1 and BM 14-3-3 proteins and NHE-1 mRNA was significantly lower by 240, 116, and 95%, respectively, in the IHEXE-Veh group compared to the IH-Veh group (all *p* < 0.05; Figures 7A–C). Compared to IH-Veh, cariporide decreased the myocardial expression of NHE-1 protein by 62%, and BM 14-3-3 protein and NHE-1 mRNA by 67% in the IH group (all *p* < 0.05; Figures 7A–C). However, there were no significant differences between the CON-Cari and CON-Veh groups, the EXE-Cari and EXE-Veh groups and the IHEXE-Cari and IHEXE-Veh groups (all *p* > 0.05; Figures 7A–C).

## Discussion

Our study showed that IH-induced increased myocardial oxidative stress, causing NHE-1 hyperactivation, subsequently contributing to accumulation fibrotic tissue accumulation in heart sections. Notably, exercise training attenuated IH-induced cardiac fibrosis, similar to the beneficial NHE-1 blocking effect of cariporide, resulting in cardiac fibrosis suppression.

Cardiomyocyte disarray and fibrosis were reportedly observed after 1 week of long-term intermittent hypobaric hypoxia (LTIHH)-normobaric normoxia and more severe fibrosis after 2 weeks of LTIHH (Lin et al., 2008). Moreover, exposure to IH for 4 weeks induced cardiac hypertrophy, cardiac fibrosis, cardiac inflammation, and even cardiac dysfunction in rats. In addition to the above changes, exposure to chronic IH for 8 weeks induced cardiac apoptosis, oxidative stress and damage, along with cardiac dysfunction, with progressive pathological changes (Yin et al., 2012). Fibrosis accumulation may involve transforming growth factor-beta (TGF-β) pathway activation with increased fibronectin expression (Ares-Carrasco et al., 2009). In hearts, TGF-β mediates effects on fibroblast proliferation and collagen synthesis in the myocardium. Activated TGF-β will suppress tissue inhibitors of metalloproteinases (TIMPs) that increase activation of matrix metalloproteinases (MMPs; Seeland et al., 2002). MMP-2 and MMP-9 have been shown to release extracellular matrix-bound latent TGF-β, thereby inducing collagen synthesis (Fan et al., 2012). One of our previous studies suggested that 12 days of IH induced increases in myocardial protein levels of MMP-2 (Chen et al., 2015). Similarly, a higher ratio of mRNA levels of MMP-2/TIMP-2 was reportedly involved with chronic IH-induced increase in cardiac fibrosis in the left ventricle (Ding et al., 2014). Conversely, activation of TGF-β signaling can upregulate MMP-2 and MMP-9 expression (Kim et al., 2004), which is a major contributor to fibrosis and matrix remodeling and is amplified by increases in oxidative stress (Hagler et al., 2013). In this study, exposure to IH for 14 days caused increased myocardial levels of lipid and protein peroxidation and decreased catalase and total SOD activities, similar to previous studies (Chen et al., 2011a,b; Williams et al., 2010). Therefore, our findings, IH-induced increased myocardial expression of TGF-β and oxidative stress concomitant with lower levels of TIMP-1 and higher levels of MMP-9 protein, collagen-1 mRNA and fibronectin mRNA and cardiac fibrosis, suggest IH-induced increase in cardiac fibrosis in rat hearts.

It has been reported that enhanced activity of NHE-1 increases intracellular Na^+^ with a consequent rise in Ca^2+^ through the Na^+^/Ca^2+^ exchanger, leading to cardiac dysfunction, cardiac hypertrophy and heart failure (Cingolani and Ennis, 2007). However, NHE-1 blockade has been shown to provide beneficial cardioprotective effects against myocardial ischemia and post-infarction (Young and Funder, 2003). In addition, blocking NHE-1 activity, either pharmacologically or by a transgenic knockdown approach, significantly reduced pro-inflammatory microglial activation, and pro-inflammatory cytokine formation in ischemic brains (Shi et al., 2011). The inhibition of redox-sensitive kinase p90 (p90^RSK^) and NHE-1 phosphorylation could alleviate the severity and improve cardiac function in patients with chronic heart disease (Cingolani et al., 2011). In this study, we assayed the myocardial expression levels of a 14-3-3 binding motif (BM 14-3-3) as an indicator of NHE-1 activity. Because p90^RSK^ phosphorylates NHE-1 at Ser703, this creates a binding site for 14-3-3 proteins (Cingolani et al., 2011). Our findings showed that IH induced increased levels of BM 14-3-3 phosphorylation, which represented high NHE-1 activity, and was inhibited by the NHE-1 blocker cariporide. These results suggest that IH-induced NHE-1 hyperactivity plays a role in IH-induced cardiac fibrosis.

Interestingly, inhibition of NHE-1 has been shown to abolish intracellular pH regulation and reduce the production of the superoxide anion and the expression of cytokines and inducible nitric oxide synthase (Liu et al., 2010). In addition, cariporide significantly resists the decrease in nitric oxide content and SOD activity, and elevation of MDA caused by high glucose (Wang et al., 2005) and homecysteine (Wu et al., 2013) in aortic tissues. Similarly, our findings showed that cariporide intervention attenuated MDA and protein carbonyl concentrations and enhanced catalase and SOD activities in rat hearts exposed to IH. In addition, the regulatory capacity of NHE-1 was improved by 4 weeks of strenuous exercise training (Feger and Starnes, 2013b), contributing to increased myocardial NHE1 activity at physiological pH, which would likely enhance cardiac performance under physiological conditions (Feger and Starnes, 2013a). One of our previous studies showed that 5 consecutive days of moderate exercise could improve myocardial antioxidant capacity and lower myocardial levels of NHE-1 protein, 24 h after the last bout of exercise (Chen et al., 2015). Although, in this study, 10 weeks of exercise induced increased myocardial antioxidant capacity, NHE-1 expression was not significantly decreased. This is probably associated with extracellular regulated protein kinase (ERK1/2) mediating NHE-1 activation (Feger and Starnes, 2013b). Once activated, ERK1/2 phosphorylates p90^RSK^, increasing NHE-1 activation (Cingolani et al., 2011). Moreover, ERK1/2 plays a major role in H_2_O_2_-induced NHE-1 phosphorylation (Snabaitis et al., 2002). Activation of ERK1/2 in the hearts of 4-week exercise-trained rats was increased after a 30-min bout of exercise but was unchanged in the 8- and 12-week-trained rats; however, there was no significant difference in ERK1/2 activation in the heart between sedentary and 4-, 8-, and 12-week-trained rats in the resting state (Iemitsu et al., 2006).

On the other hand, citrate synthase has been extensively used as a metabolic marker in assessing oxidative and respiratory capacity in skeletal muscles. It is generally recognized that the skeletal muscle oxidative capacity and mitochondrial enzyme activity are elevated by endurance exercise training (Siu et al., 2003). Therefore, increased citrate synthase activity indicates that the animals were aerobically trained due to the exercise protocol (Thorp et al., 2007). However, no appreciable alteration in citrate synthase enzyme activity was observed in the ventricular muscles of trained rats (Siu et al., 2003). Herein, a significantly higher level of citrate synthesis activity in the soleus muscle was found in rats exercise for 10 weeks, corroborating previous studies reporting significantly increased citrate synthase activity at 8 (Siu et al., 2003) and 14 weeks (Thorp et al., 2007) of exercise training. These results suggest that the exercise protocol induced adaptive effects of training. Therefore, lower levels of cardiac fibrosis in rats with 14 days of IH combined with 10 weeks of exercise and cariporide intervention suggest that regular exercise training yielded similar inhibitory effects on NHE-1 to regulate cardiac NHE-1 activation.

### Clinical application and significance

It has been well-established that the IH pattern of hypoxia–reoxygenation is an accepted animal model to mimic the physiological and pathological consequences in OSA patients (Fletcher, 2000; Lai et al., 2006). OSA is a risk factor for LV dysfunction (Chen et al., 2015), hypertension (Zhao et al., 2012; Hu et al., 2015; Meng et al., 2016) and heart disease (Dergacheva et al., 2014). To avoid the development of heart disease, OSA patients are treated with nasal continuous positive airway pressure (CPAP), which is the gold standard treatment, in order to improve cardiac function (Beker et al., 2014). Moreover, it has been reported that IH plays a role in cardiac fibrosis, which contributes to cardiac dysfunction in OSA animal models (Lin et al., 2008; Li et al., 2015). Therefore, understanding the effects of exercise on IH-induced NHE-1-mediated cardiac fibrosis should help to develop exercise-based therapeutic strategies in OSA patients. A previous study suggested that regular, moderate-intensity exercise (60 min per day, 3 days per\for 4 months) decreases OSA severity and sleep-disordered breathing in patients with OSA (Chen et al., 2011b; Ueno et al., 2009). Therefore, our findings suggest that regular, moderate exercise might lower the risk of developing cardiac fibrosis in OSA patients.

### Limitations

Three IH patterns of intermittent hypoxia are established in animals to mimic sleep apnea (hypoxic episodes lasting 10–40 s), chronic lung disease (hypoxic episodes can last as long as minutes–hours) and periodic types of breathing (periods of hyperventilation are followed by apneas lasting several seconds–minutes). The first pattern of recurrent apnea is the most clinically relevant; therefore, there is more experimental information on this paradigm (Prabhakar, 2001). Therefore, a different IH pattern might have induced different levels of myocardial NHE-1 activity, resulting in different cardiac fibrosis. Moreover, the heart response to anti-fibrosis effects through exercise is quite different depending upon the exercise protocols, which might provide different anti-fibrosis effects. Despite these limitations, our data suggest that these differences are physiologically relevant and reflect the normal variation that occurs during *in vivo* IH insults in humans with regular moderate exercise (Chen et al., 2011b).

### Conclusions

In conclusion, IH-induced cardiac fibrosis is associated with NHE-1 hyperactivity. However, exercise training exerted an inhibitory effect to prevent myocardial NHE-1 hyperactivity, which contributed to reduced IH-induced cardiac fibrosis. Therefore, NHE-1 plays a critical role in the effect of exercise in IH-induced increased cardiac fibrosis.

## Author contributions

Conceived and designed the experiments: T-IC. Performed the experiment: T-IC, W-CT. Contributed reagents/materials/analysis tools: T-IC. Analyzed the data: T-IC, W-CT. Wrote the manuscript: T-IC. All authors read and approved the final manuscript.

### Conflict of interest statement

The authors declare that the research was conducted in the absence of any commercial or financial relationships that could be construed as a potential conflict of interest.

## References

[B1] Ares-Carrasco S. Picatoste B. Benito-Martin A. Zubiri I. Sanz A. B. Sanchez-Nino M. D.. (2009). Myocardial fibrosis and apoptosis, but not inflammation, are present in long-term experimental diabetes. Am. J. Physiol. Heart Circ. Physiol. 297, H2109–H2119. 10.1152/ajpheart.00157.2009 19820199

[B2] Baguet J. P. Barone-Rochette G. Tamisier R. Levy P. Pépin J. L. (2012). Mechanisms of cardiac dysfunction in obstructive sleep apnea. Nat. Rev. Cardiol. 9, 679–688. 10.1038/nrcardio.2012.141 23007221

[B3] Beker F. Rogerson S. R. Hooper S. B. Wong C. Davis P. G. (2014). The effects of nasal continuous positive airway pressure on cardiac function in premature infants with minimal lung disease: a crossover randomized trial. J. Pediatr. 164, 726–729. 10.1016/j.jpeds.2013.10.087 24345453

[B4] Besse S. Tanguy S. Boucher F. Le Page C. Rozenberg S. Riou B.. (2004). Cardioprotection with cariporide, a sodium-proton exchanger inhibitor, after prolonged ischemia and reperfusion in senescent rats. Exp. Gerontol. 39, 1307–1314. 10.1016/j.exger.2004.06.006 15489053

[B5] Chen M. Y. C. Yang K. T. Shen Y. J. Cheng C. F. Tu W. C. Chen T. I. (2015). Role of sodium-hydrogen exchanger-1 (NHE-1) in the effect of exercise on intermittent hypoxia-induced left ventricular dysfunction. Chin. J. Physiol. 58, 254–262. 10.4077/CJP.2015.BAE357 26211649

[B6] Chen T. I. Lai C. J. Hsieh C. J. Tsai K. L. Yang K. T. (2011a). Differences in left ventricular cardiomyocyte loss induced by chronic intermittent hypoxia between spontaneously hypertensive and Wistar-Kyoto rats. Sleep Breath. 15, 845–854. 10.1007/s11325-010-0448-y 21136300

[B7] Chen T. I. Shen Y. J. Wang I. C. Yang K. T. (2011b). Short-term exercise provides left ventricular myocardial protection against intermittent hypoxia-induced apoptosis in rats. Eur. J. Appl. Physiol. 111, 1939–1950. 10.1007/s00421-010-1824-9 21249391

[B8] Cingolani H. E. Ennis I. L. (2007). Sodium-hydrogen exchanger, cardiac overload, and myocardial hypertrophy. Circulation 115, 1090–1100. 10.1161/CIRCULATIONAHA.106.626929 17339567

[B9] Cingolani O. H. Pérez N. G. Ennis I. L. Alvarez M. C. Mosca S. M. Schinella G. R.. (2011). *In vivo* key role of reactive oxygen species and NHE-1 activation in determining excessive cardiac hypertrophy. Pflugers Arch. 462, 733–743. 10.1007/s00424-011-1020-8 21870055

[B10] Dergacheva O. Weigand L. A. Dyavanapalli J. Mares J. Wang X. Mendelowitz D. (2014). Function and modulation of premotor brainstem parasympathetic cardiac neurons that control heart rate by hypoxia-, sleep-, and sleep-related diseases including obstructive sleep apnea. Prog. Brain Res. 212, 39–58. 10.1016/B978-0-444-63488-7.00003-3 25194192

[B11] Ding W. X. Dong Y. B. Ding N. Zhang X. F. Zhang S. J. Zhang X. L.. (2014). Adiponectin protects rat heart from left ventricular remodeling induced by chronic intermittent hypoxia via inhibition of TGF-beta/smad2/3 pathway. J. Thorac. Dis. 6, 1278–1284. 10.3978/j.issn.2072-1439.2014.07.44 25276370 PMC4178112

[B12] Fan D. Takawale A. Lee J. Kassiri Z. (2012). Cardiac fibroblasts, fibrosis and extracellular matrix remodeling in heart disease. Fibrogenesis Tissue Repair 5:15. 10.1186/1755-1536-5-15 22943504 PMC3464725

[B13] Feger B. J. Starnes J. W. (2013a). Exercise alters the regulation of myocardial Na+/H+ exchanger-1 activity. Am. J. Physiol. Regul. Integr. Comp. Physiol. 305, R1182–R1189. 10.1152/ajpregu.00228.2013 24049114

[B14] Feger B. J. Starnes J. W. (2013b). Myocardial Na+/H+ exchanger-1 (NHE1) content is decreased by exercise training. J. Physiol. Biochem. 69, 305–312. 10.1007/s13105-012-0214-7 23055051

[B15] Filgueiras-Rama D. Arias M. A. Iniesta A. Armada E. Merino J. L. Peinado R.. (2013). Atrial arrhythmias in obstructive sleep apnea: underlying mechanisms and implications in the clinical setting. Pulm. Med. 2013:426758. 10.1155/2013/426758 23691306 PMC3649713

[B16] Fletcher E. C. (2000). Cardiovascular consequences of obstructive sleep apnea: experimental hypoxia and sympathetic activity. Sleep, 23(Suppl. 4), S127–S131. 10893085

[B17] Guo J. Gan X. T. Haist J. V. Rajapurohitam V. Zeidan A. Faruq N. S.. (2011). Ginseng inhibits cardiomyocyte hypertrophy and heart failure via NHE-1 inhibition and attenuation of calcineurin activation. Circ. Heart Fail. 4, 79–88. 10.1161/CIRCHEARTFAILURE.110.957969 20971938

[B18] Hagler M. A. Hadley T. M. Zhang H. Mehra K. Roos C. M. Schaff H. V.. (2013). TGF-β signalling and reactive oxygen species drive fibrosis and matrix remodelling in myxomatous mitral valves. Cardiovasc. Res. 99, 175–184. 10.1093/cvr/cvt083 23554457 PMC3687751

[B19] Hu W. Jin X. Gu J. Zhang P. Yu Q. Yin G.. (2015). Risk factor panels associated with hypertension in obstructive sleep apnea patients with different body mass indexes. J. Am. Soc. Hypertens. 9, 382–389. 10.1016/j.jash.2015.01.015 25766498

[B20] Iemitsu M. Maeda S. Jesmin S. Otsuki T. Kasuya Y. Miyauchi T. (2006). Activation pattern of MAPK signaling in the hearts of trained and untrained rats following a single bout of exercise. J. Appl. Physiol. (1985) 101, 151–163. 10.1152/japplphysiol.00392.2005 16484365

[B21] Karmazyn M. Kilic A. Javadov S. (2008). The role of NHE-1 in myocardial hypertrophy and remodelling. J. Mol. Cell Cardiol. 44, 647–653. 10.1016/j.yjmcc.2008.01.005 18329039

[B22] Kim E. S. Kim M. S. Moon A. (2004). TGF-β-induced upregulation of MMP-2 and MMP-9 depends on p38 MAPK, but not ERK signaling in MCF10A human breast epithelial cells. Int. J. Oncol. 25, 1375–1382. 10.3892/ijo.25.5.1375 15492828

[B23] Kolarova J. D. Ayoub I. M. Gazmuri R. J. (2005). Cariporide enables hemodynamically more effective chest compression by leftward shift of its flow-depth relationship. Am. J. Physiol. Heart Circ. Physiol. 288, H2904–H2911. 10.1152/ajpheart.01181.2004 15708960

[B24] Lai C. J. Yang C. C. Hsu Y. Y. Lin Y. N. Kuo T. B. (2006). Enhanced sympathetic outflow and decreased baroreflex sensitivity are associated with intermittent hypoxia-induced systemic hypertension in conscious rats. J. Appl. Physiol. (1985) 100, 1974–1982. 10.1152/japplphysiol.01051.2005 16484362

[B25] Li J. Zhang H. Zhu W. Z. Yu Z. Guo A. Yang H. T.. (2007). Preservation of the pHi during ischemia via PKC by intermittent hypoxia. Biochem. Biophys. Res. Commun. 356, 329–333. 10.1016/j.bbrc.2007.02.128 17359938

[B26] Li W. Yan S. Zhao J. Ding X. Zhang S. Wang D.. (2015). Metoprolol Inhibits cardiac apoptosis and fibrosis in a canine model of chronic obstructive sleep apnea. Cell Physiol. Biochem. 36, 1131–1141. 10.1159/000430284 26113294

[B27] Lin Y. M. Huang S. K. Wang H. F. Chen L. M. Tsai F. J. Hsu H. H.. (2008). Short-term versus long-term intermittent hypobaric hypoxia on cardiac fibrosis and Fas death receptor dependent apoptotic pathway in rat hearts. Chin. J. Physiol. 51, 308–316. 19175187

[B28] Liu Y. Kintner D. B. Chanana V. Algharabli J. Chen X. Gao Y.. (2010). Activation of microglia depends on Na+/H+ exchange-mediated H+ homeostasis. J. Neurosci. 30, 15210–15220. 10.1523/JNEUROSCI.3950-10.2010 21068326 PMC3010222

[B29] Liu Y. Zhu H. Su Z. Sun C. Yin J. Yuan H.. (2012). IL-17 contributes to cardiac fibrosis following experimental autoimmune myocarditis by a PKCbeta/Erk1/2/NF-κB-dependent signaling pathway. Int. Immunol. 24, 605–612. 10.1093/intimm/dxs056 22531062

[B30] Meng F. Ma J. Wang W. Lin B. (2016). Obstructive sleep apnea syndrome is a risk factor of hypertension. Minerva Med. 107, 294–299. 27163297

[B31] Prabhakar N. R. (2001). Oxygen sensing during intermittent hypoxia: cellular and molecular mechanisms. J. Appl. Physiol. (1985) 90, 1986–1994. 11299293 10.1152/jappl.2001.90.5.1986

[B32] Seeland U. Haeuseler C. Hinrichs R. Rosenkranz S. Pfitzner T. Scharffetter-Kochanek K.. (2002). Myocardial fibrosis in transforming growth factor-β(1) (TGF-β(1)) transgenic mice is associated with inhibition of interstitial collagenase. Eur. J. Clin. Invest. 32, 295–303. 10.1046/j.1365-2362.2002.00985.x 12027867

[B33] Shi Y. Chanana V. Watters J. J. Ferrazzano P. Sun D. (2011). Role of sodium/hydrogen exchanger isoform 1 in microglial activation and proinflammatory responses in ischemic brains. J. Neurochem. 119, 124–135. 10.1111/j.1471-4159.2011.07403.x 21797866 PMC3192493

[B34] Siu P. M. Bryner R. W. Martyn J. K. Alway S. E. (2004). Apoptotic adaptations from exercise training in skeletal and cardiac muscles. FASEB J. 18, 1150–1152. 10.1096/fj.03-1291fje 15132982

[B35] Siu P. M. Donley D. A. Bryner R. W. Alway S. E. (2003). Citrate synthase expression and enzyme activity after endurance training in cardiac and skeletal muscles. J. Appl. Physiol. (1985) 94, 555–560. 10.1152/japplphysiol.00821.2002 12531911

[B36] Snabaitis A. K. Hearse D. J. Avkiran M. (2002). Regulation of sarcolemmal Na(+)/H(+) exchange by hydrogen peroxide in adult rat ventricular myocytes. Cardiovasc. Res. 53, 470–480. 10.1016/S0008-6363(01)00464-3 11827698

[B37] Thorp D. B. Haist J. V. Leppard J. Milne K. J. Karmazyn M. Noble E. G. (2007). Exercise training improves myocardial tolerance to ischemia in male but not in female rats. Am. J. Physiol. Regul. Integr. Comp. Physiol. 293, R363–R371. 10.1152/ajpregu.00363.2006 17507436

[B38] Ueno L. M. Drager L. F. Rodrigues A. C. Rondon M. U. Braga A. M. Mathias W.. (2009). Effects of exercise training in patients with chronic heart failure and sleep apnea. Sleep 32, 637–647. 19480231 10.1093/sleep/32.5.637 PMC2675899

[B39] Wang S. X. Xiong X. M. Song T. Liu L. Y. (2005). Protective effects of cariporide on endothelial dysfunction induced by high glucose. Acta Pharmacol. Sin. 26, 329–333. 10.1111/j.1745-7254.2005.00042.x 15715929

[B40] Williams A. L. Chen L. Scharf S. M. (2010). Effects of allopurinol on cardiac function and oxidant stress in chronic intermittent hypoxia. Sleep Breath. 14, 51–57. 10.1007/s11325-009-0279-x 19603215

[B41] Wu S. Gao X. Yang S. Liu L. Ge B. Yang Q. (2013). Protective effects of cariporide on endothelial dysfunction induced by homocysteine. Pharmacology 92, 303–309. 10.1159/000356318 24296950

[B42] Xue J. Mraiche F. Zhou D. Karmazyn M. Oka T. Fliegel L.. (2010). Elevated myocardial Na+/H+ exchanger isoform 1 activity elicits gene expression that leads to cardiac hypertrophy. Physiol. Genomics 42, 374–383. 10.1152/physiolgenomics.00064.2010 20460605 PMC2929882

[B43] Yin X. Zheng Y. Liu Q. Cai J. Cai L. (2012). Cardiac response to chronic intermittent hypoxia with a transition from adaptation to maladaptation: the role of hydrogen peroxide. Oxid. Med. Cell Longev. 2012:569520. 10.1155/2012/569520 22685619 PMC3364002

[B44] Young M. Funder J. (2003). Mineralocorticoid action and sodium-hydrogen exchange: studies in experimental cardiac fibrosis. Endocrinology 144, 3848–3851. 10.1210/en.2003-0039 12933657

[B45] Zhao Q. Liu Z. H. Luo Q. Zhao Z. H. Zhang H. L. Wang Y. (2012). Effects of continuous positive airway pressure on blood pressure and daytime sleepiness in obstructive sleep apnea patients with coronary heart diseases under optimal medications. Sleep Breath. 16, 341–347. 10.1007/s11325-011-0498-9 21337116

